# Various cross-linking methods inhibit the collagenase I degradation of rabbit scleral tissue

**DOI:** 10.1186/s12886-020-01751-z

**Published:** 2020-12-14

**Authors:** Konstantin Krasselt, Cornelius Frommelt, Robert Brunner, Franziska Georgia Rauscher, Mike Francke, Nicole Körber

**Affiliations:** 1 Paul-Flechsig-Institute of Brain Research, Leipzig University, Liebigstraße 19, 04103 Leipzig, Germany; 2 Fachbereich SciTec, Ernst-Abbe-Hochschule Jena, University of Applied Sciences, Carl-Zeiß-Promenade 2, 07745 Jena, Germany; 3 Institute for Medical Informatics, Statistics and Epidemiology (IMISE), Leipzig University, Härtelstraße 16-18, 04107 Leipzig, Germany

**Keywords:** Scleral cross-linking, Rabbit sclera, Glutaraldehyde, Paraformaldehyde, Riboflavin/UV-A-light, Riboflavin/blue light

## Abstract

**Background:**

Collagen cross-linking of the sclera is a promising approach to strengthen scleral rigidity and thus to inhibit eye growth in progressive myopia. Additionally, cross-linking might inhibit degrading processes in idiopathic melting or in ocular inflammatory diseases of the sclera. Different cross-linking treatments were tested to increase resistance to enzymatic degradation of the rabbit sclera.

**Methods:**

Scleral patches from rabbit eyes were cross-linked using paraformaldehyde, glutaraldehyde or riboflavin combined with UV-A-light or with blue light. The patches were incubated with collagenase I (MMP1) for various durations up to 24 h to elucidate differences in scleral resistance to enzymatic degradation. Degraded protein components in the supernatant were detected and quantified using measurements of Fluoraldehyde o-Phthaldialdehyde (OPA) fluorescence.

**Results:**

All cross-linking methods reduced the enzymatic degradation of rabbit scleral tissue by MMP1. Incubation with glutaraldehyde (1%) and paraformaldehyde (4%) caused nearly a complete inhibition of enzymatic degradation (down to 7% ± 2.8 of digested protein compared to control). Cross-linking with riboflavin/UV-A-light reduced the degradation by MMP1 to 62% ± 12.7 after 24 h. Cross-linking with riboflavin/blue light reduced the degradation by MMP1 to 77% ± 13.5 after 24 h. No significant differences could be detected comparing different light intensities, light exposure times or riboflavin concentrations.

**Conclusions:**

The application of all cross-linking methods increased the resistance of rabbit scleral tissue to MMP1-degradation. Especially, gentle cross-linking with riboflavin and UV-A or blue light might be a clinical approach in future.

## Background

Cross-linking of the sclera is a promising approach to strengthen the scleral rigidity to inhibit eye growth in progressive myopia [1, 2]. The suggested beneficial effect of scleral cross-linking (SXL) treatment is the proposed reduction of the axial length elongation of highly myopic eyes to prevent pathological myopia. The risk of pathological outcomes such as chorio-retinal degeneration, retinal tears, and detachments might be minimized [3–5]. Different treatments using chemical cross-linkers or photosensitizers in combination with light of different wavelengths are object of various studies [6–8]. Cross-linking with riboflavin and UV-A or blue light increases the scleral biomechanical rigidity in a dose-dependent manner [1, 9]. Animal studies show an inhibitory effect on eye growth in rabbit [6, 7, 10]. There are differences in efficacy to strengthen the biomechanical stiffness of collagenous tissue by different cross-linkers, which were shown by stress-strain material tests of porcine corneas. According to Spoerl & Seiler [11], Karnovsky’s solution and glutaraldehyde are the most effective cross-linkers followed by riboflavin/UV-A-light, methylglyoxal, glyceraldehyde, riboflavin/blue light and others. The most ineffective cross-linkers are glucose and short-wave UV-A-light (265 nm) [11].

These findings led to the idea of treating corneal melting diseases due to collagenosis like rheumatoid arthritis [12]. Enzymes like collagenases and other matrix metalloproteinases play a key role in the pathogenesis of this kind of diseases by weakening the collagenous stroma and thus lead to thinning and deformation of the tissue structure [13, 14]. Therefore, the effect of cross-linking on the resistance to enzymatic degradation was tested by Spoerl, Wollensak and Seiler at the porcine cornea [12]. The tissue was cross-linked by riboflavin and 3 mW/cm^2^ UV-A-light (370 nm, 30 min) and degraded with pepsin, trypsin and collagenase. Total enzymatic degradation of the cornea took at least twice the time of untreated control corneas.

Variations in genes coding for metalloproteinases were detected in people being affected by myopia [15–17]. In pathological myopia, the biomechanical weakening of the sclera leads to a disproportion of the optical apparatus and an elongation of the eye globe accompanied with severe pathological outcomes [3, 4, 18]. The crucial role of active collagen remodeling in the development and recovery of myopia in tree shrew was proofed by McBrien et al. [19]. Here, the underlying mechanism is a change in metalloproteinase activity which evokes a scleral weakening in several animal models with induced myopia [20–22].

The obvious consideration is to strengthen the connective tissue fibers to prevent immoderate enzymatic degradation and thus to stop resulting thinning and deformation of the sclera. It needs to be examined if cross-linking is a suitable application to increase the resistance of scleral tissue to enzymatic degradation in myopia development and melting diseases like scleritis. Therefore, we degraded rabbit scleral tissue by collagenase I after application of different cross-linking procedures. A detection system using Fluoraldehyde o-Phthaldialdehyde (OPA) reagent was established to measure the amount of cleaved protein products (mostly collagen) in the supernatant. We compared the potency of different cross-linking methods to increase the scleral resistance to enzymatic degradation.

## Methods

### Specimen preparation

**Fig. 1:**
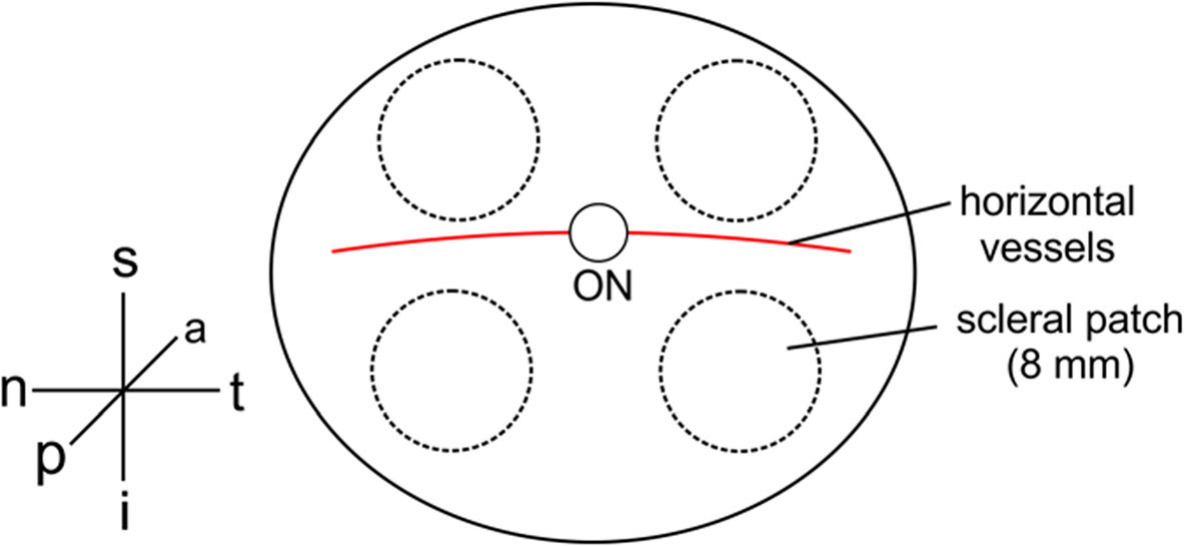
Preparation of scleral patches from the rabbit eye for enzymatic degradation. After isolation of the eye, the cornea, lens, vitreous body and inner eye layers were removed and four 8 mm patches were punched out of the sclera. ON - optic nerve, a - anterior, i - inferior, n – nasal, p – posterior, s – superior, t – temporal

**Fig. 2:**
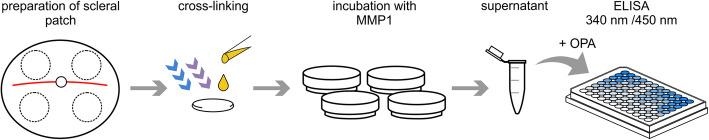
Scheme of the methodical procedure. The rabbit scleral patches were cross-linked using glutaraldehyde, paraformaldehyde, riboflavin and UV-A or blue light, respectively. The patches were incubated in 1 mg collagenase I (MMP1) for 24 h in a humid chamber at room temperature. Supernatant was taken at different time points and checked for their peptide content using Fluoraldehyde o-Phthaldialdehyde (OPA). The relative fluorescence was detected by a microplate reader (excitation 340 nm/ emission 450 nm). If the cross-linking treatment increases the scleral resistance to MMP1-degradation the relative fluorescence of the supernatant is decreased because of its lesser peptide amount compared to the untreated control

The authors have considered all ethical aspects of the study and followed the guidelines of the Helsinki Declaration and all experiments were done in accordance with the European Communities Council Directive 86/609/EEC and the ARVO Statement for the Use of Animals in Ophthalmic and Vision Research. Experiments were approved by the local authorities (Faculty of Medicine of the University of Leipzig). We did not perform any animal experiments. The albino rabbit eyes were directly obtained from a local abattoir and frozen at − 20 °C without additional fluid within 2 h post mortem. All rabbits were at an age of 12 up to 14 weeks at the time of death. The frozen albino rabbit eyes were thawed in phosphate buffered saline (PBS, pH 7.4, Biochrom AG, Berlin, Germany). Eyes were used up to 1 year post mortem. Altered or damaged eyes were discarded. After removal of outer ocular muscle and connective tissue the eye ball was opened at the ora serrata. The cornea, lens and vitreous body were removed. The eye cup was bisected in the nasal and temporal hemisphere and cleaned from retina and choroid. 8 mm patches were punched out of the sclera (Fig. 1) and weighed using an accuracy weighing machine (Mettler-Toledo GmbH, Gießen, Germany). Patch weights were 21.77 mg ± 0.6 (*n* = 88) in average. The complete methodical procedure from the specimen preparation to the peptide detection is schematically drawn in Fig. 2.

### Cross-linking with glutaraldehyde and paraformaldehyde

Scleral patches were incubated in 1% glutaraldehyde or 4% paraformaldehyde in PBS for 1 or 5 min and then rinsed in PBS for 24 h for a complete clearance of the chemical cross-linkers to ensure that these substances do not interfere with the enzyme (MMP1) itself and therefore, might inhibit the activity of the enzyme directly by protein cross-linking.

### Cross-linking with riboflavin and UV-A/blue light

Scleral patches were incubated in riboflavin (Vitamin B2, 0.5% or 0.1% in PBS without any Dextran admixture, Streuli Pharma, Uznach, Switzerland) at room temperature for 20 min. Then, the scleral pieces were irradiated with 40 or 75 mW/cm^2^ UV-A-light (365 nm ± 10 nm) or with 40 or 150 mW/cm^2^ blue light (450 ± 25 nm) for 10 min on each side. A UV-A-light source (UV-X™ 1000; IROC Innocross AG, Switzerland) was modified to provide higher light intensities. Blue light was applied using the bluephase 16i (Ivoclar Vivadent GmbH, Ellwangen-Jagst, Germany). Riboflavin was refreshed every 5 min for each patch to avoid drying of the specimen and photo-bleaching of the fluorophore. The adjustment of the applied blue light intensity (40 and 150 mW/cm^2^) was realized by custom-built polypropylene spacing tubes in the front of the irradiation device. Different UV-A-light intensities (40 and 75 mW/cm^2^) were adjusted in changing the distance of the scleral patches to the light source. The light intensities were measured and controlled by a power-meter device (LaserMate Q, Coherent Inc., Santa Clara, CA, USA). All samples including the controls were kept in PBS (pH 7.4, Biochrom AG, Berlin, Germany) while preparing the remaining specimens of the eye.

### Enzymatic degradation

The scleral patches were incubated in 3 ml extracellular solution composed of 136 mM NaCl, 3 mM KCl, 10 mM HEPES and 11 mM Glucose (Carl ROTH GmbH + Co. KG, Karlsruhe, Germany), of 1 mM MgCl_2_ (Sigma-Aldrich, Taufkirchen, Germany and 2 mM CaCl_2_ (Merck KGaA, Darmstadt, Germany), respectively. Collagenase I was added (MMP1 from Clostridium histolyticum, Sigma-Aldrich, Taufkirchen, Germany) with a concentration of 1 mg/ml up to 24 h in a humid chamber on a shaker at room temperature for enzymatic degradation. Various time intervals of 1, 4, 8 and 24 h were chosen for an enzyme kinetic analysis. At each time point 100 μl of the supernatant was extracted and stored at 4 °C for peptide detection. The supernatant volume loss was considered and mathematically corrected for the peptid analysis.

### Peptide detection

**Fig. 3:**
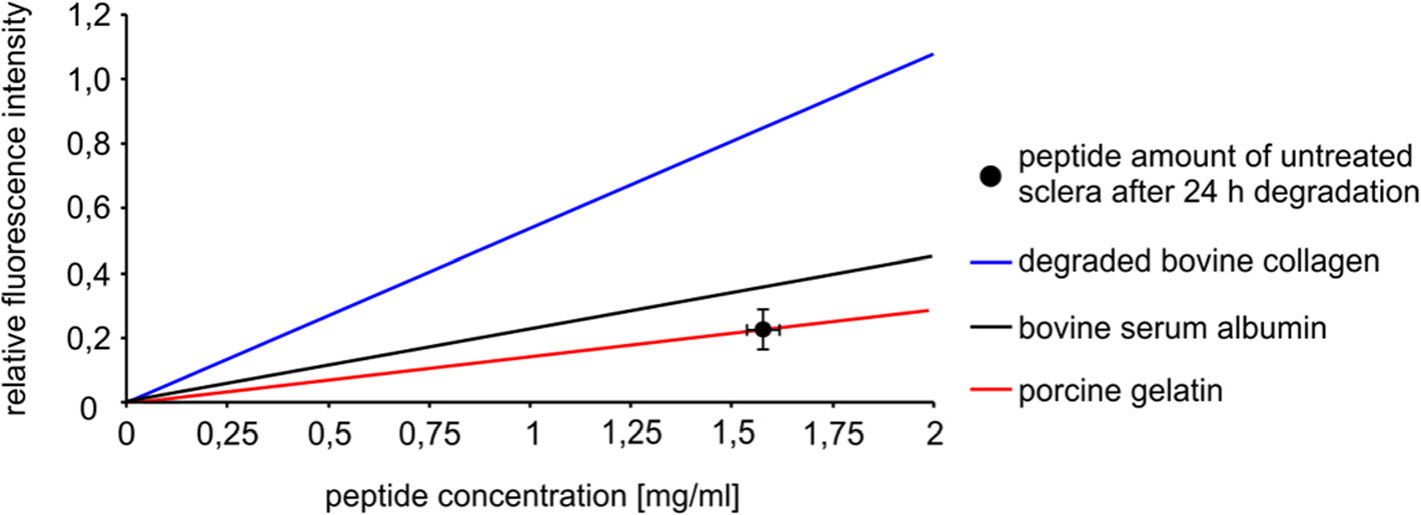
Comparison of different peptide standards for the calculation of scleral peptide degradation products. Collagenase I (MMP1) degraded bovine collagen (blue curve), bovine serum albumin (black curve) and porcine gelatin (red curve) were diluted, incubated with Fluoraldehyde o-Phthaldialdehyde and checked for their fluorescence intensity. The standard curves (based on fluorescence values of each indicated peptide concentration) were compared to the fluorescence intensity of the supernatant of non-cross-linked rabbit scleras after the 24 h-degradation by MMP1 (black dot). The peptide amount per 1 ml supernatant from the non-cross-linked sclera after 24 h degradation by MMP1 was 1.58 mg ± 0.04 (horizontal SD) with a relative fluorescence intensity of 0.229 ± 0.06 (vertical SD), *n* = 15. The means and its corresponding standard deviation are displayed. Porcine gelatin standard curve corresponds mostly to the real protein amount of the scleral patches

For analysis of degradation-products the Thermo Scientific Pierce Fluoraldehyde o-Phthaldialdehyde (OPA) Reagent (Thermo Scientific Fisher Inc., Rockford, IL, USA) was used. The chemical reacts with primary amines of amino acids, peptides and proteins to a fluorescent product that can be detected by fluorescence measurements (excitation/emission = 340 nm/455 nm). A calibration curve was determined using a standard aqueous solution of bovine serum albumin (BSA, 2 mg/ml, Thermo Scientific Fisher Inc., Rockford, IL, USA), degraded bovine collagen (degraded by 1 mg/ml MMP1 in PBS + Glucose, from achilles tendon, Sigma-Aldrich, Taufkirchen, Germany) and porcine gelatine (from porcine skin, Sigma -Aldrich, Taufkirchen, Germany), respectively. All proteins were checked for their value as a fitting standard (Fig. 3). For calculation a reference value for the patches the dry weight of the scleral patches was determined by weighting the patches before and after a complete drying period with a precise analytical balance. The protein amount of the scleral patches was estimated from the total patch weight (21.77 mg ± 0.6) minus 78.3% water content (own unpublished data).

Twenty μl peptide solution / supernatant was mixed with 200 μl Fluoraldehyde OPA solution and incubated for 10 min for fluorescence measuring as recommended by the manufacturer and each sample was measured three times to identify outlier. Fluorescence was measured by an ELISA-Reader (anthos htIII, anthos Mikrosysteme GmbH, Krefeld, Germany) using the 340 nm excitation filter and the 450 nm emission filter. The relative fluorescence of the incubation medium (extracellular solution + MMP1) was set as blank and subtracted from all other values. The amount of peptide in the supernatant was calculated as percentage of the degradation products of the corresponding control sclera after 24 h in each experiment. The corresponding control values were incubated without any cross-linking procedure. The values were averaged and illustrated using Microsoft® Office Excel 2003 (© 1985–2003 Microsoft Corporation). Significances were calculated with Origin Pro 2017G SR1 (© OriginLab Corporation) using the Mann-Whitney U test (unpaired, two-tailed).

## Results

### Comparison of different peptide standards

The quantification of a peptide amount in the supernatant extinguished from the relative fluorescence intensity requires an eligible standard protein. The comparison of three peptides showed different standard curves after incubation with Fluoraldehyde o-Phthaldialdehyde (Fig. 3). MMP1 degraded bovine collagen (blue curve, y = 0.537x + 0.0023) showed stronger fluorescence intensities in comparison to bovine serum albumin (black curve, y = 0.2239x + 0.0025) and porcine gelatin (red curve, y = 0.1447x – 0.0041).

The supernatant peptide amount of the untreated (that means no cross-linking) rabbit sclera after a 24 h-degradation by MMP1 (black dot in Fig. 3) was measured to estimate the value of each of these standard curves. Bovine serum albumin and porcine gelatin standard curves showed similarity and can both be used to calculate the real peptide amount in the supernatant after degradation by MMP1. Because of the good solubility, easy handling and the daily use in our laboratories we used bovine serum albumin as standard for all experiments.

### Macroscopic appearance of the scleral patches after collagenase I degradation

**Fig. 4:**
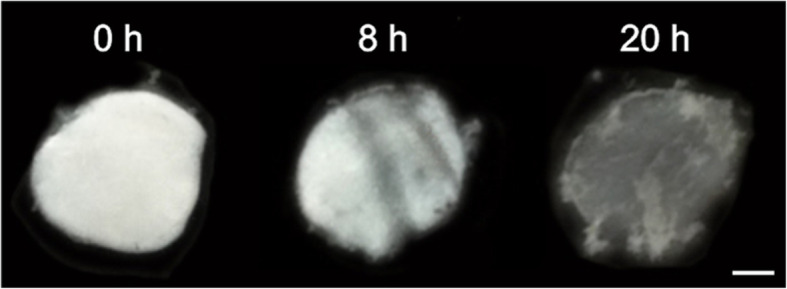
Optical appearance of untreated (non-cross-linked) rabbit scleral patches after 0, 8 and 20 h MMP1-degradation (after 24 h barely visible). No remarkable decrease of scleral patch diameter could be observed. The tissue became transparent and the surface became rough. Small tissue/protein clots are dissociated from the patch and finally, a translucent tissue network remained in the incubation solution at 24 h. Scale bar: 2 mm

Figure 4 shows exemplary the optical appearance of scleral patches after 1, 8 and 20 h of degradation by MMP1. An obvious decrease of the scleral patch diameter could not be observed. Over a digestion-time up to 24 h the tissue became transparent and the surfaces became rough. Small protein aggregates and tissue clots dissociated from the scleral patch appearing in the incubation medium. Finally, a translucent network of the former patch remained in the solution. Even after 72 h of incubation with collagenase I a residual network derived from scleral tissue remains and no increase of the peptide amount in the supernatant compared to 24 h incubation could be detected.

### MMP1 degradation of untreated (non-cross-linked) sclera

**Fig. 5:**
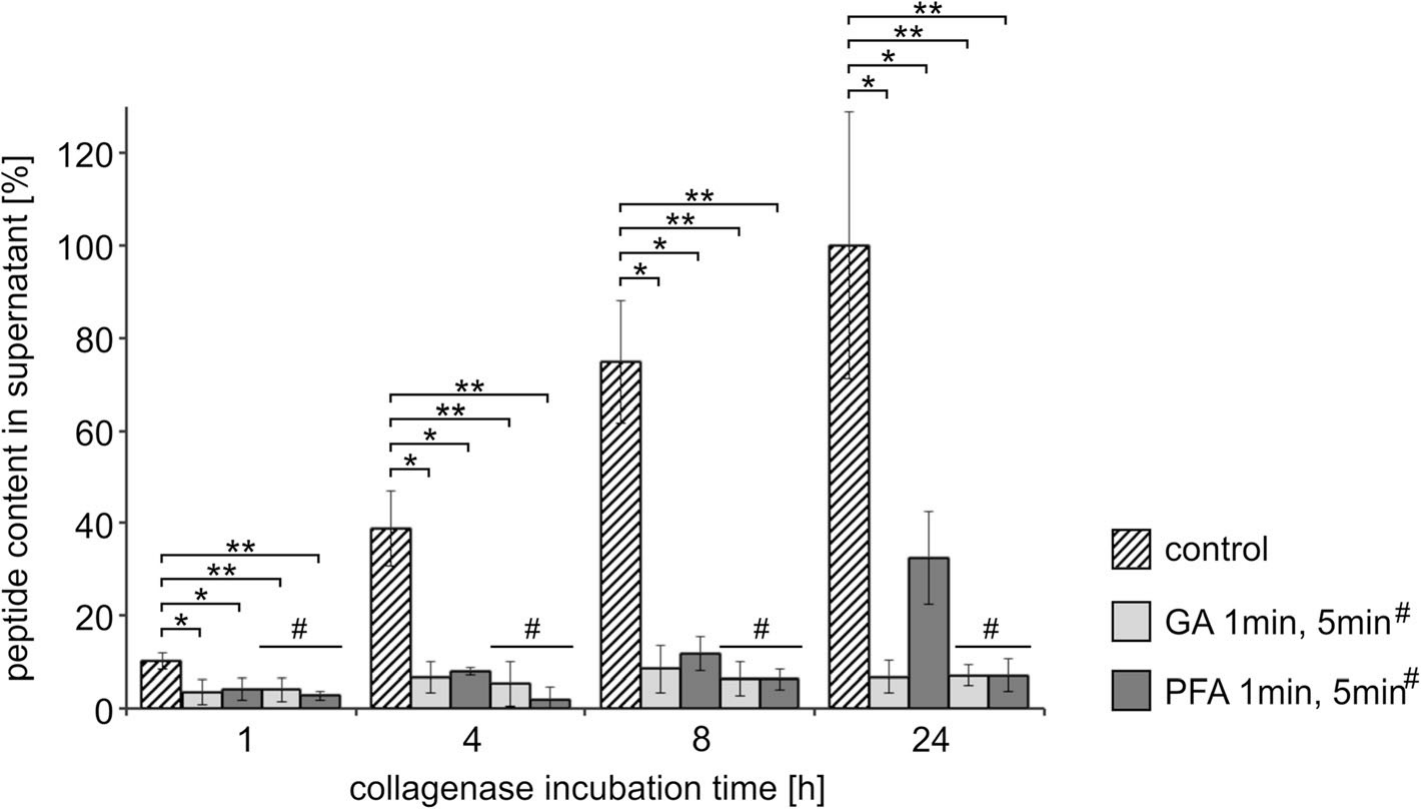
Resistance of rabbit sclera to MMP1-degradation after cross-linking with glutaraldehyde (GA) and paraformaldehyde (PFA). Scleral patches were incubated (“fixed”) for 1 or 5 min (# indicates the 5 min values in the diagram) in GA and PFA. The amount of peptide in the supernatant was calculated as percentage of the degradation products of the untreated (non-cross-linked) control value after 24 h (each 24 h value of the corresponding internal control patch was set 100%). GA and PFA 1 min *n* = 3; GA and PFA 5 min and all non-crosslinked controls *n* = 5. Means and standard deviations are presented. The statistical analysis was performed with the Mann-Whitney U-Test (unpaired, two tailed), p* < 0.05, p** < 0.01

**Fig. 6:**
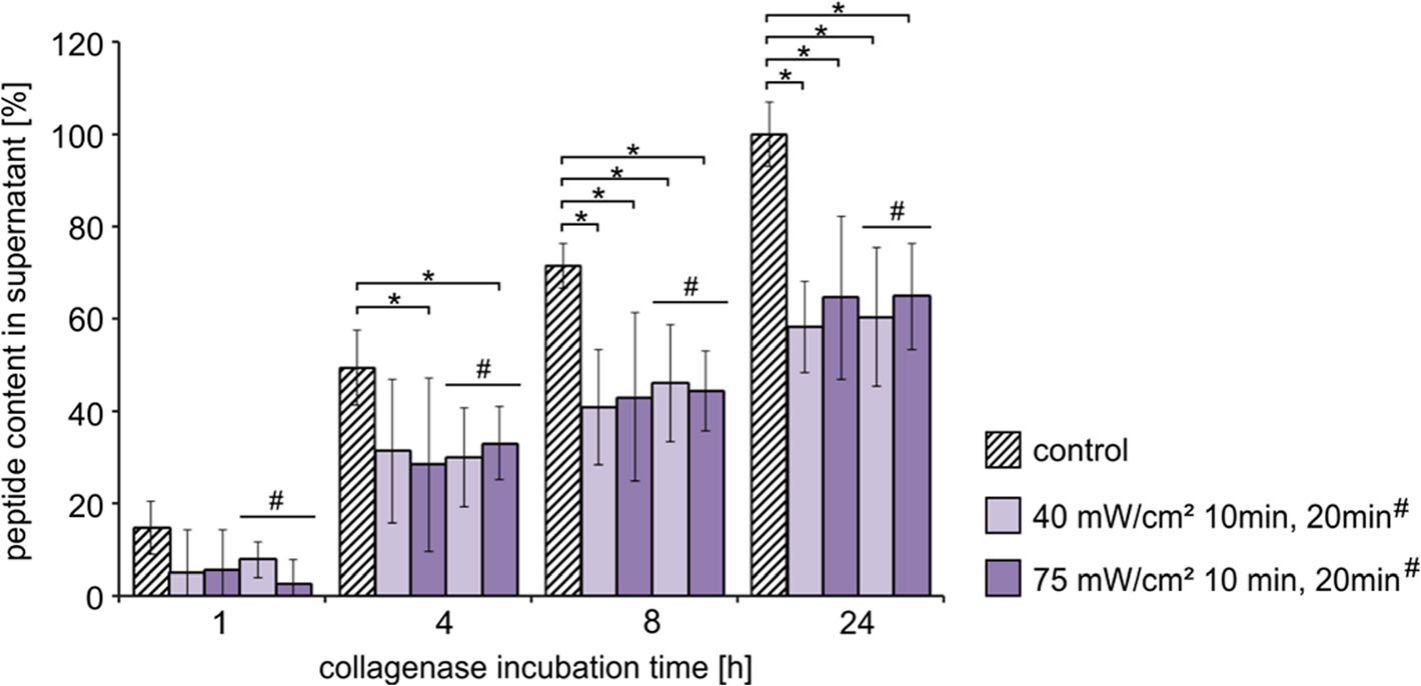
Resistance of rabbit sclera to MMP1-degradation after cross-linking with riboflavin and UV-A-light with different light intensities (0.5% riboflavin combined with 40 or 75 mW/cm^2^) and different light exposure times (10 or 20 min (# indicate the 20 min time in the diagram)). The amount of peptide in the supernatant was calculated as percentage of the degradation products of the untreated (non-cross-linked) control value after 24 h (each 24 h value of the corresponding internal control patch was set 100%). Means (*n* = 4) and standard deviation are presented. The statistical analysis was performed with the Mann-Whitney U-Test (unpaired, two tailed), p* < 0.05

**Fig. 7:**
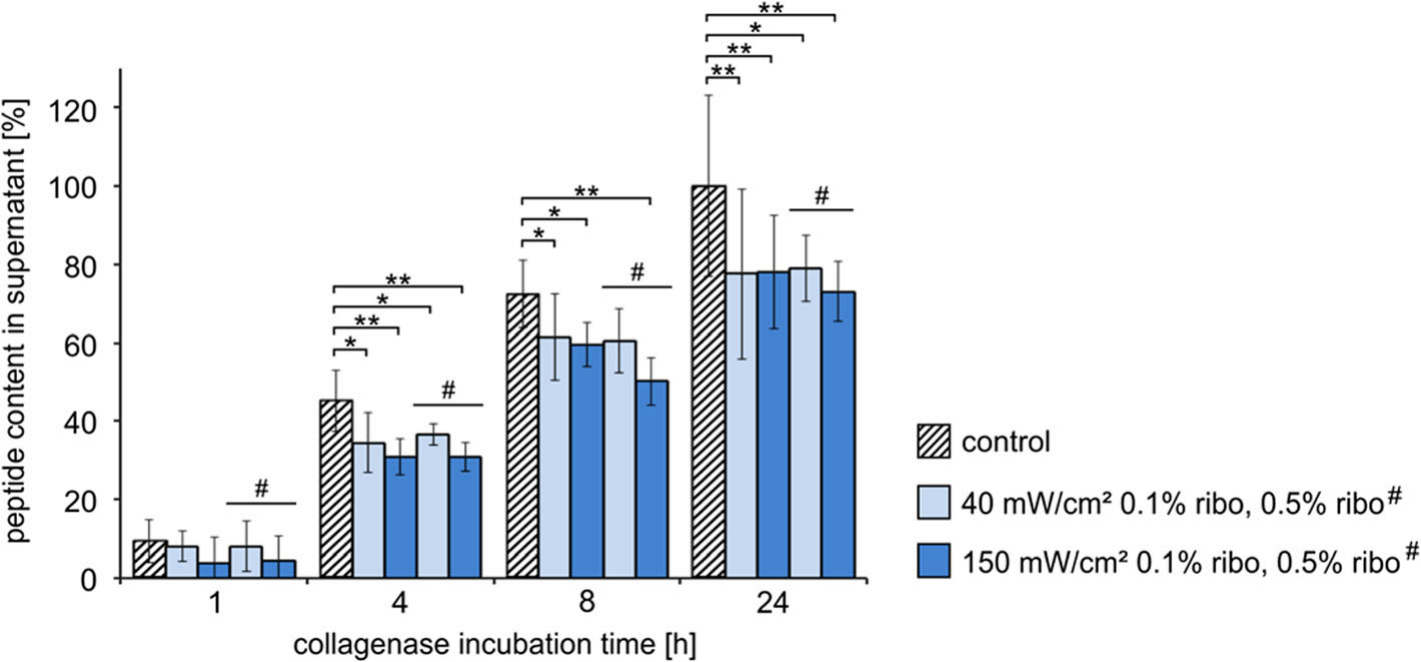
Resistance of rabbit sclera to MMP1-degradation after cross-linking with riboflavin and blue light of different light intensities (20 min exposure time with 40 or with 150 mW/cm^2^) and treatment with different riboflavin concentrations (0.1% or 0.5% (# indicate the treatment with 5% riboflavin in the diagram). The amount of peptide in the supernatant was calculated as percentage of the degradation products of the untreated (non-cross-linked) control value after 24 h (each 24 h value of the corresponding internal control patch was set 100%). Means (*n* = 6) and the standard deviations are presented. The statistical analysis was performed with the Mann-Whitney U-Test (unpaired, two tailed), p* < 0.05, p** < 0.01

In a separate series of experiments we elucidate the time dependence of the enzymatic digestion of the untreated control scleral patches. The peptide amount in the supernatant after 24 h degradation of untreated sclera patches was set 100% as the control values to calculate the degradation degree after 1, 4 and 8 h. After 1 h incubation with MMP1 10.9% ± 4.9 of the peptide compared to the untreated control sclera was detected in the supernatant. 44.2% ± 8.5 were measured at 4 h and at 8 h 73% ± 9.2 of the control peptide could be detected (mean of all controls, *n* = 15). These values indicate a nearly linear digestion process up to 7 h and after 24 h most of the control patches were completely degraded (data calculation not shown). Despite a linear enzymatic digestion up to 7 h we decide to measure all above mentioned time points of each experimental approach to ensure a measurable amount of digested protein in all supernatant samples (especially, in experiments with strong inhibition of the digestion). All internal control values of the following experiments associated with various cross-linking methods are in consistency with this separate series of control measurements to establish the methodology (compare internal controls in Figs. 5, 6 and 7).

### MMP1 degradation after cross-linking with glutaraldehyde and paraformaldehyde

Scleral patches were treated with glutaraldehyde (GA) or paraformaldehyde (PFA) for 1 or 5 min to evaluate the resulting resistance to enzymatic degradation by MMP1. Both cross-linking chemicals (“fixative agents” for biological material) significantly reduced the degradation of scleral tissue by MMP1 (Fig. 5). After 24 h only approximately 7% of the fixed patches were enzymatically degraded (GA, 1 min to 6.8% ± 3.4; GA, 5 min to 7% ± 2.3 and PFA, 5 min to 7% ± 3.5). The incubation for 1 min in PFA reduced the degradation to 32.6% ± 10 after 24 h. Therefore, GA is a stronger cross-linker and inhibitor of the enzymatic digestion than PFA after very short-time (1 min) incubation. After 5 min incubation (fixation) with GA we could not detect any remarkably differences of the enzymatic degrading potency. The enzymatic degradation was more reduced after 5 min incubation with paraformaldehyde compared to 1 min incubation (Fig. 5).

### MMP1 degradation after cross-linking with riboflavin and UV-A

The treatment with riboflavin and UV-A-light significantly reduced the enzymatic degradation of scleral tissue in comparison to the untreated control (Fig. 6). At 24 h 62% ± 12.7 of the sclera was degraded after cross-linking with riboflavin and UV-A-light compared to control. Neither clear difference of inhibition could be detected using higher light intensities nor higher light exposure times (40 mW/cm^2^/10 min to 58.1% ± 9.9 vs. 40 mW/cm^2^/20 min to 60.4% ± 17.8 and 75 mW/cm^2^/10 min to 64.5% ± 14.9 vs. 75 mW/cm^2^/20 min to 64.9% ± 11.5). The treatment of the scleral tissue with riboflavin (0.5%) only without an UV-A or blue light irradiation did not result in a decelerated enzymatic sclera degradation (data not shown).

### MMP1 degradation after cross-linking with riboflavin and blue light

Cross-linking with riboflavin and blue light reduced the scleral degradation to 76.9% ± 13.5 after 24 h (Fig. 7). Neither clear difference of inhibition could be detected using higher light intensities nor a higher riboflavin concentration (40 mW/cm^2^/0.1% riboflavin to 77.7% ± 14.6 vs. 40 mW/cm^2^/0.5% riboflavin to 79% ± 21.7 and 150 mW/cm^2^/0.1% riboflavin to 78% ± 7.7 vs. 150 mW/cm^2^/0.5% riboflavin to 73.1% ± 8.5).

### Comparison of glutaraldehyde, paraformaldehyde, riboflavin/UV-A-light and riboflavin/blue light

**Fig. 8:**
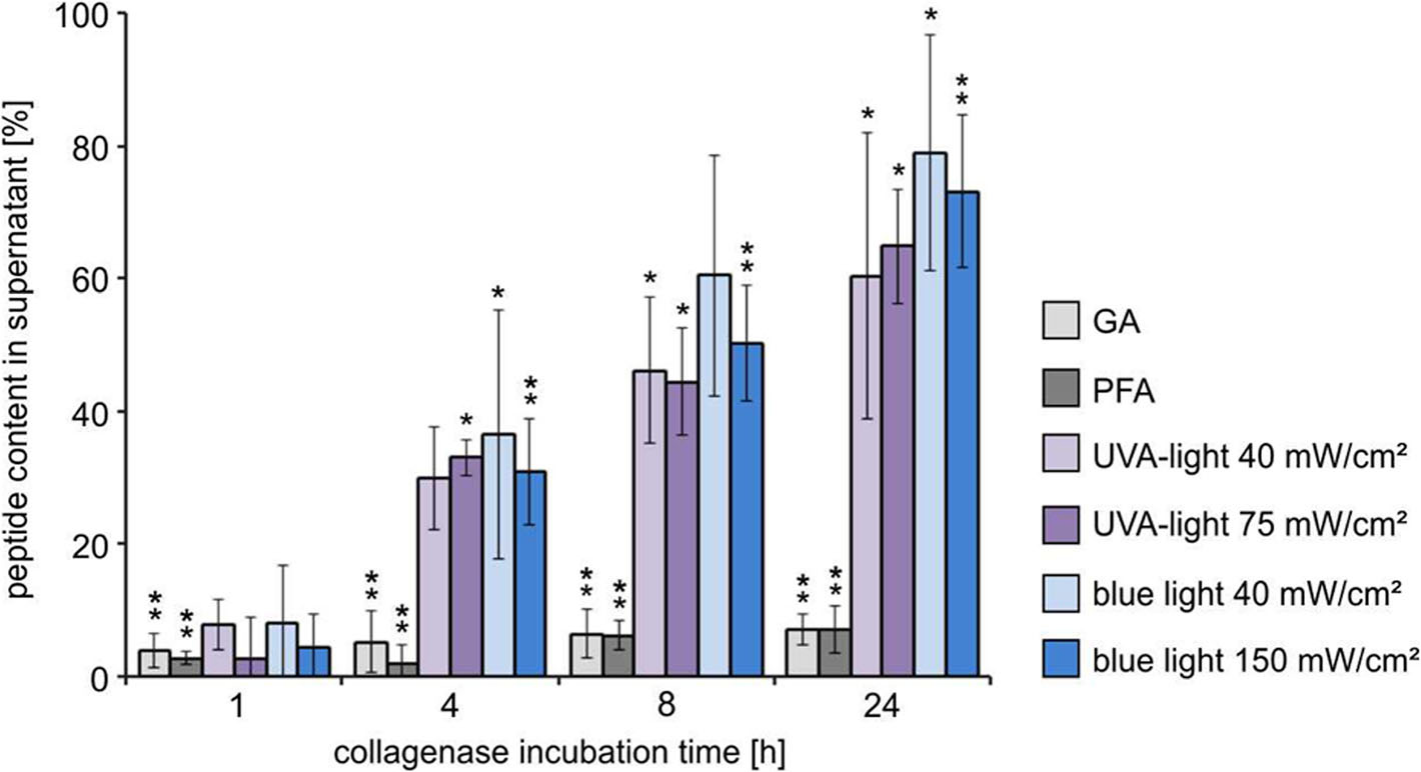
Comparison of rabbit scleral resistance to MMP1 after cross-linking with glutaraldehyde (GA, 1%, 5 min), paraformaldehyde (PFA, 4%, 5 min), riboflavin/UV-A-light (0.5% riboflavin; 40 and 75 mW/cm^2^, 20 min irradiation time) and riboflavin/blue light (0.5% riboflavin; 40 and 150 mW/cm^2^, 20 min irradiation time) of different light intensities. The amount of peptide in the supernatant was calculated as percentage of the degradation products of the untreated (non-cross-linked) control value after 24 h (each 24 h value of the corresponding internal control patch was set 100%). Each of the cross-linking methods reduced the degradation of rabbit sclera by MMP1. Cross-linking with the chemical cross-linkers GA and PFA reduced the degradation to 7% at 24 h (*n* = 5). The treatment with riboflavin/UV-A-light reduced the enzymatic degradation of rabbit sclera to 62% at 24 h (*n* = 4). No significant differences in resistance to enzymatic degradation were detected using higher light intensities. After riboflavin/blue light cross-linking the enzymatic degradation of rabbit sclera was reduced to 77% at 24 h (*n* = 6). No differences in resistance to enzymatic degradation were detected using higher light intensities. Means and standard deviation are presented. The statistical analysis was performed with the Mann-Whitney U-Test to the corresponding control (unpaired, two tailed), p* < 0.05, p** < 0.01

The direct comparison of differently cross-linked sclera (Fig. 8) shows that the chemical cross-linking with glutaraldehyde and paraformaldehyde is most effective with a significantly higher reduction of enzymatic degradation down to 7% ± 2.8 compared to the cross-linking with riboflavin and UV-A or blue light.

The resistance to enzymatic digestion seems to be stronger in scleral tissue treated with riboflavin and UV-A-light compared to a treatment with riboflavin and blue light. The treatment of scleral tissue with riboflavin and UV-A-light reduced the enzymatic degradation to 62% ± 12.7 whereas the treatment with riboflavin and blue light caused a reduction to 76.9% ± 13.5 (24 h values). However, the differences were not statistically significant probably caused by the restricted numbers of experiments combined with the biological variation.

In this study the treatment with riboflavin and blue light has the smallest impact as cross-linker and shows the lowest effect on inhibition of the enzymatic degradation. Neither changes of riboflavin concentration nor changes of the light intensities of each wavelength effects the outcome significantly. Obviously, the grade of inhibition of the enzymatic degradation at every time point (1, 4, 8 and 24 h) in each of the examined cross-linking approaches is similar.

## Discussion

Our study revealed for the first time, that all examined cross-linking approaches increase the resistance to enzymatic digestion of rabbit scleral tissue, at which the chemical cross-linkers were the most effective. Therefore, cross-linking methods might be in future a promising clinical approach to treat ocular inflammatory or melting diseases of the eye.

Spoerl, Wollensak and Seiler evaluated the enzymatic resistance of porcine corneas by measuring the decreasing diameter after incubation with collagen degrading enzymes [12]. Contrary, in our experiments the macroscopic appearance of the scleral patch samples (as shown in Fig. 3) did not enable such quantification of degradation by collagenase 1 (MMP1). We did not observe a remarkable decrease of the scleral patch diameter instead of other changes as sign of tissue degradation (e.g. tissue becomes transparent).

This might be due to a slightly different biochemical composition of the rabbit scleral tissue compared to the porcine cornea and the specificity of the used collagenase I enzyme. The extracellular matrix of the sclera is composed of fibrillary proteins such as collagen and elastin and additionally of amorphous ground substance such as proteoglycans and glycoproteins. Type I, III, V and VI collagen are present in the sclera, although biochemical analyses have shown that collagen type I predominates the composition while type III represent less than 5% additionally with a small amount of other protein species [23]. MMP1 is specific to collagen I, II and III (and some other collagens), but cleaves also gelatin, some other extracellular matrix proteins and serum proteins. However, it does not cleave elastin [24]. This may explain a residual network of non-degraded elastin, proteoglycans and glycoproteins of the scleral remaining after 24 h incubation with MMP1. Thus, measuring of patch diameters was not an appropriate method for studying the scleral resistance to enzymatic degradation.

For that reason, we decided to measure the scleral collagen degradation products in the supernatant with a sensitive peptide detection method. Since 1971 the reagent o-Phthaldialdehyde (OPA) + 2-mercaptoethanol is used to detect even small amounts of amino acids, peptides and proteins. Its high sensitivity in the picomole range is due to its reaction with primary amines yielding highly fluorescent isoindole derivatives [25, 26]. GO et al. [27] showed that OPA could detect cleaved collagen (enzymatically degraded collagen, detection of > 5 μg) with a linear relationship between collagen amount and fluorescence emission. The exposure of the N-termini resulting from collagen breakage leads to an increase of the OPA fluorescence [27]. The exposure of collagen to bacterial collagenase increased the fluorescence intensity in a dependence of increasing enzyme amounts. In comparison to mammalian collagenase, the bacterial collagenase cleaves collagen in small peptides and not only to ¾ and ¼-fractions [28]. That increases the sensitivity. Consequently, OPA is a suitable and simple realizable method to detect bacterial MMP1-degradation products of collagen.

In future, methods to change the resistance to enzymatic degradation should also be examined with other collagen cleaving enzymes like gelatinase A, stromelysin and gelatinase B which are expressed by scleral fibroblasts [29]. These results might provide an indication of several types of cross-links generated by different methods and their cross-linking strength.

### Cross-linking by GA and PFA

In our study, we found that all cross-linking methods reduced the enzymatic degradation by MMP1 of rabbit scleral tissue.

The exposure of proteins to various cross-linkers is accompanied by generation of inter- and intramolecular covalent bonds [30, 31]. Cross-linking increases the resistance of collagenous tissue to enzymatic degradation by collagenase I because the tertiary structure of the protein is changed and with that, the enzymatic access to specific cleavage sites is hampered [13, 32].

In this study, the chemicals glutaraldehyde (GA) and paraformaldehyde (PFA) are the strongest cross-linkers with a nearly complete inhibition of the enzymatic degradation. GA and PFA form bonds between their aldehyde groups and adjacent macromolecules and remain as molecule in the tissue [33–35]. With a 5 min incubation/fixation time, GA and PFA similarly reduced the enzymatic degradation of scleral tissue. This result confirms earlier studies, which showed a similar degree of bacterial collagenase I degradation of collagen after a glutaraldehyde or formaldehyde treatment [36, 37]. However, the chemical cross-linking mechanism of both is different [38] and fixation by formaldehyde (compared to glutaraldehyde) is a slower and generates cross-links with time [39, 40]. This might explain our result, that one-minute incubation with PFA reduced the enzymatic degradation only to 33%.

### Cross-linking by riboflavin and UV-A or blue light

In contrast to chemical cross-linkers, the bonding of collagen fibrils by a physical cross-linking is mediated by reactive oxygen species and free radicals. These have a single unpaired electron, which makes them highly reactive against surrounding substances [41], and thereby, can connect amino residues of adjacent collagen molecules. No additional chemical groups are incorporated in the covalent cross-links between amino acids of the collagen molecules in comparison to chemical cross-linkers as glutaraldehyde or formaldehyde [42].

The physical cross-linking with the photosensitizer riboflavin and irradiation with light of different wavelengths reduced the MMP1-degradation rate of scleral tissue to 62% with UV-A-light and 77% with blue light. This difference might be explainable by the shorter wavelength of UV-A-light compared to blue light. Short-wavelength light possesses more photon energy and might lead to a stronger production of reactive oxygen species and free radicals when applied to excitable molecules [43, 44]. Furthermore, UVA light irradiation of riboflavin induces similar reactive oxygen species, radicals and chemical decay products as blue light but in other proportions [45]. Thus, the application of UV-A-light and riboflavin lead to other amounts of reactive oxygen species and free radicals and therefore, to an increased total amount of cross-links in scleral tissue compared to blue light of comparable light intensity (e.g. 40 mW/cm^2^). Consequently, the enzymatic digestion is more affected by a riboflavin/UV-A-light application than after riboflavin/blue light application. This phenomenon was also observed comparing different cross-linking technics of their ability to increase the biomechanical stiffness of porcine corneas. According to Spoerl & Seiler, Karnovsky’s solution and glutaraldehyde are the most effective cross-linkers followed by riboflavin/UV-A-light, methylglyoxal, glyceraldehyde, riboflavin/blue light and ribose. The weakest cross-linkers are glucose and short-wave UV-A-light (265 nm) [11]. The usage of riboflavin with UV-A-light led to a two-fold higher increase in corneal stiffness compared to the application of riboflavin and blue light at same exposure times and light intensities [11].

No significant differences in resistance to enzymatic degeneration could be detected when comparing different light intensities, light exposure times and riboflavin concentrations (Figs. 6, 7 and 8). Spoerl et al. could identify clear differences in enzymatic resistance after usage of 1 and 2 mW/cm^2^ UV-A-light in porcine corneas at which a higher light intensity resulted in a stronger resistance [12]. As well as longer irradiation time (30 and 45 min) with UV-A-light led to a stronger increase of the biomechanical rigidity of porcine corneas [11]. In our studies, the differences might be too small to be distinguished with the performed method or the applied light intensities (40 and 75 mW/cm^2^ UV-A-light; 40 and 150 mW/cm^2^ blue light) were too high so that the maximal cross-link amount was already achieved. That means, we were in a saturation range with the lowest intensity and irradiation time applied. To elucidate the detailed kinetic of cross-linking an extended series of experiments must be performed in future.

Since riboflavin is not only the provider of reactive oxygen species but also a scavenger itself, a balance between reactive oxygen species generation and elimination is reached at higher concentrations. Therefore, an increase in riboflavin concentration does not inevitably lead to a higher yield of reactive oxygen species [46]. This might explain why we could not detect any differences in enzymatic resistance of scleral tissue treated with 0.1 and 0.5% riboflavin and blue light. In future, variations of concentration, light intensities and irradiation times should be probed to characterize parameters for the most effective and gentle application in humans to strengthen the sclera in melting diseases such as scleritis or in progressive myopia.

## Conclusion

Scleral tissue degradation occurs in ocular inflammatory or melting diseases of the eye. Our study revealed for the first time, that cross-linking treatments with glutaraldehyde, paraformaldehyde or riboflavin application combined with UV-A-light or blue light irradiation inhibit the enzymatic degradation of scleral tissue. Although, the chemical cross-linkers were the most effective in inhibiting enzymatic degradation the clinically established procedures of riboflavin application combined with UV-A-light or blue light are the promising approaches for a future clinical treatment.

## Data Availability

The datasets analyzed in this study are available from the corresponding author (Mike Francke, mike.francke@medizin.uni-leipzig.de) upon reasonable request.

## Abbreviations

GA: Glutaraldehyde
MMP1: Matrix-Metallo-Proteinase 1 (i.e. collagenase I)
OPA: Ortho-Phthaldialdehyde
PBS: Phosphate buffered saline
PFA: Paraformaldehyde
SXL: Scleral cross-linking
UV-A-light: Ultraviolet light A
